# SPARC Negatively Correlates With Prognosis After Transarterial Chemoembolization and Facilitates Proliferation and Metastasis of Hepatocellular Carcinoma via ERK/MMP Signaling Pathways

**DOI:** 10.3389/fonc.2020.00813

**Published:** 2020-05-21

**Authors:** Yao Liu, Ying Feng, Xiaojing Wang, Xue Yang, Ying Hu, Yuxin Li, Qun Zhang, Yunyi Huang, Ke Shi, Chongping Ran, Jie Hou, Li Jiang, Junfa Li, Xianbo Wang

**Affiliations:** ^1^Center of Integrative Medicine, Beijing Ditan Hospital, Capital Medical University, Beijing, China; ^2^Department of Gastroenterology, Dongzhimen Hospital, Beijing University of Chinese Medicine, Beijing, China; ^3^Department of Surgery, Beijing Ditan Hospital, Capital Medical University, Beijing, China; ^4^Department of Neurobiology and Center of Stroke, Beijing Institute for Brain Disorders, Capital Medical University, Beijing, China

**Keywords:** hepatocellular carcinoma, transarterial chemoembolization, secreted protein acidic and rich in cysteine, extracellular signal-regulated kinase, matrix metalloproteinase

## Abstract

**Background:** Transarterial chemoembolization (TACE) represents a widely accepted treatment procedure for intermediate stage or unresectable hepatocellular carcinoma (HCC). However, few studies have evaluated serologic prognosis factors in patients with HCC before TACE. Secreted protein acidic and rich in cysteine (SPARC) is a matricellular glycoprotein affecting tumorigenesis and metastasis, and leading to poor prognosis in HCC. Therefore, to further explore the potential prognosis value of SPARC, the expression levels in the plasma of patients and its potential molecular mechanisms underlying the regulation of HCC were investigated in this study.

**Materials and Methods:** The study population included 43 patients with HCC who underwent TACE. To evaluate the expression of SPARC in different grades of pathological tissues, the immunohistochemistry was performed on tissues from 89 patients with HCC. Lentiviral vectors carrying interference sequences, as well as vectors harboring the complete open reading frame of SPARC for the knockdown or overexpression of SPARC in HuH-7 or HepG2 cells, respectively, allowed us to determine the biological functions of SPARC *in vitro* and *in vivo*. We also evaluated the levels of phosphorylated extracellular signal-regulated kinases 1/2 (p-ERK1/2) and matrix metalloproteinases 2/9 (MMP2/9) activation.

**Results:** The association between serum levels of SPARC and the survival at different TNM and Barcelona-Clinic Liver Cancer (BCLC) stages in patients with HCC undergoing TACE were evaluated. We observed a significant upregulation of SPARC in high grade HCC tissues, predicting unfavorable prognosis, and suggesting an important tumor-promoting effect of SPARC. Functional studies indicated that downregulation of SPARC contributed to the inhibition of proliferation and metastasis of HuH-7 cells *in vitro*, whereas its overexpression led to opposite phenotypes. Mechanistically, decreased expression of SPARC resulted in dephosphorylation of ERK1/2 and deactivation of MMP2/9, thereby inhibiting growth and metastasis of HCC. Importantly, low expression levels of SPARC inhibited the formation of subcutaneous tumors in nude mice.

**Conclusions:** SPARC was found to facilitate proliferation and metastasis of HCC via modulation of the ERK1/2-MMP2/9 signaling pathways. Our research has provided a glimpse on the biological mechanism of SPARC and might contribute to the eventual treatment of liver cancer.

## Introduction

Hepatocellular carcinoma (HCC) constitutes the fifth most common malignancy and the third cause of cancer-related mortality worldwide (1). The 5-years survival rate exhibited by patients with HCC after receiving liver transplantation, curative hepatic resection (HR), or radiofrequency ablation (RFA) of early stage tumors, has been reported to be ~70% (2, 3). However, for cases of unresectable HCC, including tumors with poor liver function reserve, more than three tumor nodules localized to different segments or lobes, portal vein tumor thrombosis (PVTT), or extrahepatic metastases, transarterial chemoembolization (TACE) has been used as the preferred treatment method of choice (4). The prognosis of patients with unresectable HCC after implementation of TACE has been reported to vary significantly. Therefore, it is of vital significance for the clinical practice to explore the mechanisms of incidence and progress of HCC after TACE, as well as identify and evaluate the underlying prognosis targets.

Secreted Protein and Rich in Cysteine (SPARC), also known as osteonectin or BM-40, is known to act as a kind of matricellular glycoprotein. There have been many reports about the special functions of SPARC, including inhibition of cell spreading (5), cell migration, angiogenesis (6), and cell cycle (7). Matrix metalloproteinases (MMPs) have been widely recognized as crucial factors for the degradation of extracellular matrix proteins, and for also facilitating the formation of distal metastases. Recently, several genes, including extracellular signal-regulated kinases 1/2 (ERK1/2) and SPARC have been associated with the modulation of the activity of MMP2/9 in HCC (8–10). However, the intermediate factor in the SPARC-induced regulation of the expression of MMP2/9 in HCC is still uncharacterized.

A previous study evaluating the correlation between SPARC level in the peritumoral-activated hepatic stellate cells and prognosis in patients with HCC after resection reported that SPARC independently contributed to high recurrence or death rates (11). However, to the best of our knowledge, there are no studies that have investigated the prognostic role of SPARC in patients with HCC, especially in patients who underwent TACE. To this end, we quantified the levels of serum SPARC and analyzed their correlation to prognosis in patients with HCC after TACE, as well as performed immunohistochemical assays to evaluate the expression of SPARC in different grades of pathological tissues.

Moreover, in this study we assessed the potential mechanism and possible signaling pathways involving SPARC during the development of HCC. The ability of SPARC to promote the proliferation and metastasis of cells both *in vitro* and *in vivo* was assessed via downregulating or upregulating the expression of SPARC in HuH-7 and HepG2 cells, respectively. This antitumor effect could be attributed to the inhibition of the ERK/MMPs pathway, which has been shown to be important in the regulation of HCC proliferation and metastasis.

## Materials and Methods

### Selection of Patients and Clinical Samples

The diagnosis of HCC was performed according to the criteria of the American Association for Liver Diseases Study (12). The study involved 43 patients receiving initial TACE treatment at the Beijing Ditan Hospital, Capital Medical University from April 2014 to July 2015. The inclusion criteria of the study were as follows: (1) patients with HCC at Barcelona-Clinic Liver Cancer (BCLC) stages of A, B, or C, patients with HCC at Tumor Node Metastasis (TNM) stages of II, III, or IVA, and patients with Child-Pugh class A or B; (2) patients who suffered extrahepatic metastasis; (3) patients with HBV etiologies. Whereas, exclusion criteria were: (1) patients suffering hepatitis A, D, or E, autoimmune liver disease, syphilis, and AIDS; (2) patients whose clinical data were incomprehensive and had insufficient follow-up. To evaluate the expression of SPARC in different grades of pathological tissues, we performed immunohistochemical assays in hepatic tissues collected from 89 patients with HCC.

### Transarterial Chemoembolization Procedure

Before chemoembolization, superior mesenteric angiography, and common hepatic angiography were performed to assess tumor vascularity, vascular anatomy, and tumor range. After administering local anesthesia to patients, the Seldinger technique was adopted to introduce a 5F catheter into the abdominal aorta via the superficial femoral artery. During hepatic arterial angiography, fluoroscopy assisted in introducing the catheter into the celiac and superior mesenteric arteries, followed by identification of the feeding artery and staining of the tumor and of the surrounding vascular anatomy. A microcatheter was introduced into the feeding artery via the catheter. Then, 5–10 mL of ultra-fluid lipiodol, 20–40 mg of lobaplatin, and 10–30 mg of pirarubicin were combined and subsequently introduced into the tumor. If there was a significant arterioportal (AP) shunt, it was necessary to embolize the gelatin sponge particles to occlude the shunt. Additional angiography was performed before completing the operation to ensure that the supplying artery was fully blocked.

### Follow-Up

The study took the overall survival (OS) and progression-free survival (PFS) of patients as the endpoints, which were measured from the time of the initial diagnosis of HCC to the death or the last follow-up date of patients. During the period of follow-up, those with recurrent symptoms, such as recurrence of local lesions, extrahepatic recurrence, as well as intrahepatic distant recurrence received conservative treatment, TACE, or other targeted treatments based on the status of their liver function and the features exhibited by the recurrent tumor.

### Immunohistochemistry

Tissue sections fixed with formalin and embedded with paraffin were deparaffinized using a graded series of alcohol washes, and then subjected to 1 h of antigen retrieval and blockage using 5% bovine serum albumin (BSA). Tissue sections were then incubated with antibodies against SPARC (1:200, Abcam, Cambridge, UK), p-ERK1/2, and total ERK1/2 (1:400, CST, Danvers, MA, USA), MMP-2 (1:100, CST), and MMP-9 (1:150, CST) at 4°C overnight. After washing, the secondary goat anti-mouse IgG (ZSGB-BIO, Beijing, China) was added and incubated for 1 h at room temperature. The tissue sections were stained with 3,3′-diaminobenzidine (ZSGB-BIO, Beijing, China) and hematoxylin (Solarbio, Beijing, China). Next, the tissue sections were scanned in a Pannoramic MIDI scanner (3DHISTECH, Budapest, Hungary) and the images were captured using the CaseViewer software (3DHISTECH, version 2.0). The degree of immunostaining was scored separately by two independent investigators. The scores were determined by combining the proportion of positively stained tumor cells and the intensity of staining. The proportion of positively stained tumor cells was graded as follows: score 0, <5% positively stained tumor cells; score 1, 5–25% positively stained tumor cells; score 2, 26–50% positively stained tumor cells; score 3, 51–75% positively stained tumor cells; score 4, >75% positively stained tumor cells. The intensity of staining was scored on a scale of 0–3 as follows: score 0, no staining; score 1, weak staining, light yellow; score 2, moderate staining, yellowish-brown; score 3, strong staining, brown. The staining index was calculated as follows: staining index = staining intensity × proportion of positively stained tumor cells. Tumors with staining index scores of 8–12 were considered to exhibit high SPARC expression, whereas those with staining index scores of 0–7 were considered to exhibit low SPARC expression.

### Cell Lines and Infection

HCC cell lines (SMMC7721, MHCC97L, MHCC97H, BEL7402, HUH-7, and HEPG2) and the MIHA normal liver cell line cells were provided by the China Infrastructure of Cell Line Resources (Beijing, China). Cells were cultured in Dulbecco's modified Eagle's medium (DMEM) supplemented with 1% glutamine, 1% penicillin/streptomycin, and 10% fetal bovine serum (FBS). All cells were cultured at 37°C in a 5% CO_2_ atmosphere, and passaged once every 2–3 days. The SPARC-shRNA (shR), scramble-sequence (SCR), SPARC-infected (SPARC) and negative control (NC) lentiviruses containing the gene encoding the green fluorescent protein (GFP) were obtained from Gene Pharma Inc. (Shanghai, China). HuH-7 cells were cultured at 8 × 10^4^ cells/well in 6-well plates and infected with SPARC-shR and scramble-shRNA lentiviruses, following the instructions of the manufacturer. The abbreviation CON indicates the HuH-7 or HepG2 control cells (untreated HuH-7 or HepG2 cell lines). The ERK inhibitor PD98059 (20 μM, Sigma-Aldrich, Santa Clara, California, USA) was added to HuH-7 cells for 1 day. The same procedure was followed for the infection of HepG2 cells. The efficiency of the 72 h infection with the shR and SPARC lentiviruses was evaluated via Leica DM6000B microscope with 10 × 0.25 Numerical Aperture (NA) objective lens (Leica, Wetzlar, Germany).

### Real-Time Quantitative Polymerase Chain Reaction

Total RNA was extracted from transfected HepG2 and HuH-7 cells using the TRIzol reagent (Invitrogen, Carlsbad, California, USA). The obtained RNA was reverse transcribed to cDNA using the One TaqRT-PCR kit (New England Biolabs, Ipswich, MA, USA). For the quantitative reverse transcriptase polymerase chain reaction (RT-qPCR), the SuperReal qPCR PreMix (SYBR Green) (TIANGEN, Beijing, China) was used in a CFX96TM Real-Time system (BIO-RAD, Hercules, California, USA), as well as in a C1000TM Thermal Cycler (BIO-RAD, Hercules, California, USA) according to the protocols of the manufacturers. To evaluate the expression of SPARC, we designed the following primers: Forward, 5′-CCCATTGGCGAGTTTGAGAAG-3′; and Reverse, 5′-CAGGCAAGGGGGGATGTATT-3′. The expression of SPARC was normalized to the expression of the β-actin housekeeping gene.

### Subcutaneous Xenograft Nude Mice Models

BALB/c-A nude mice (4-weeks-old male) were purchased from the animal center of the Vital River (Beijing, China) and were maintained at the same center. The mice were housed (three mice per cage) in a pathogen-free environment. The mice had free access to aseptic water and food. The study protocol was approved by the Vital River Institutional Animal Care and Use Committee (permit number: RSD-SOP-002-01). All animal experiments were conducted according to the recommendations in the Guide for the Care and Use of Laboratory Animals of the National Institutes of Health. After randomly dividing them into two groups of 10 mice each, we then generated the nude mice specific subcutaneous HCC tumor model. HuH-7 cells with a stably decreased expression of SPARC or SCR were first transfected with a luciferase expressing lentivirus, and then subcutaneously injected into the armpit of nude mice (1 × 10^7^ cells). A Vernier caliper was used for measuring the growing tumor size every 3–4 days for 1 mo. Then, 30 days later, mice with subcutaneous tumors were sacrificed. Tumor tissues were surgically resected, fixed in formalin, and embedded in paraffin. Tissues embedded in paraffin were used in immunohistochemistry analyses.

### Cell Proliferation Assays

The relative proliferation capacity and ability of cells for colony formation were measured using the MTT and plate colony formation assays. HuH-7 and HepG2 cells previously infected with SCR, shR, NC, or SPARC for 24 h, were then seeded at 4,000 cells/well in 96-well plates, with every group consisting of five replicate wells. The MTT assay assisted in determining the relative proliferation of cells for 4 days. In brief, we added 20 μL of MTT solution at a concentration of 5 mg/mL to every well and incubated cells at 37°C for 4 h. Optical density was measured at 490 nm, and the number of live cells was approximated based on the absorbance values of cells solubilized in 150 μL DMSO (Sigma-Aldrich). For the plate colony formation assay, cells previously infected for 24 h, were plated in 6-well plates at a concentration of 1,000 cells/well for every experimental group. DMEM supplemented with 10% FBS was replaced every 2–3 days. After 10 days incubation, every well was washed with phosphate buffered saline (PBS) and stained with crystal violet. Colonies of cells were manually counted under Leica DM6000B microscope with 10 × 0.25 NA objective lens (Leica, Wetzlar, Germany).

### Cell Migration and Invasion Assays

For the invasion assay, we used the trans-well chamber assay with an 8 μm pore filter membrane under a pro-coating of Matrigel (BD Biosciences, Franklin Lakes, New Jersey, USA), whereas for the cell migration assay the extracellular matrix (ECM) was omitted (Corning Costar, Corning, NY, USA). HepG2 and HuH-7 cells were cultured in medium (200 μL DMEM without FBS) at a density of 1 × 10^5^ cells/upper well. The lower chamber was filled with a certain amount of complete media (600 μL DMEM containing 10% FBS). The membranes were first incubated at 37°C for 24 h. Next, the medium in the upper chamber was discarded and the cells were removed using a cotton swab. Cells were fixed with 100% alcohol and stained for 30 min with 1% crystal violet. No <6 random microscopic fields were observed under Leica DM6000B microscope with 10 × 0.25 NA objective lens (Leica, Wetzlar, Germany).

### Western Blot Analysis

The expression of proteins in cells infected by SCR, shR, SPARC, or NC was assessed via Western blot analysis. Total protein was isolated in RIPA buffer (high) with 1 mM phenylmethylsulfonyl fluoride (Solarbio, Beijing, China) for 30-min and then centrifuged at 13,000 g for 15 min at 4°C. After quantification of protein, an equal amount (40 μg) was loaded in each sample well, and separated on 15% SDS-polyacrylamide gels, followed by electrotransfer to polyvinylidene difluoride (PVDF) membranes. Membranes were incubated with anti-rabbit monoclonal antibodies against SPARC (1:400; Abcam), ERK/p-ERK (1:1000; CST), MMP-2 (1:500; CST), and MMP-9 (1:500; CST). Detection using anti-GAPDH antibodies (1:1000; CST) helped to ensure equal loading of protein samples on gels. The integrated density exhibited by protein bands was quantified using the Alpha View software (ProteinSimple, Santa Clara, California, USA).

### The Cancer Genome Atlas Data

Data regarding the levels of SPARC mRNA expression in HCC (The Cancer Genome Atlas, TCGA, Nature 2014) were extracted from cBioPortal (www.cbioportal.org).

### Statistical Analysis

The clinical and demographic characteristics in this study were summarized as median, range and number (Table 1). We converted the continuous values to categorical values considering the cut-off values calculated according to the largest Youden index (Sensitivity + Specificity −1) value. The Student's *t*-test was used to compare samples in terms of continuous variable age, while the Pearson's chi-square test was applied to compare the relationship between two or more categorical variables. Important predictive factors of the prognosis of patients with HCC receiving TACE treatment were identified using the univariate and multivariate Cox proportional hazards regression analyses. The OS and PFS of patients were analyzed with the Kaplan Meier (KM) method by virtue of the log rank test. All experiments were performed for at least 3 times. The Student's *t*-test assisted in analyzing the differences between different experimental groups regarding the expression of the SPARC RNA, the proliferation of tumor cells, the numbers of formed colonies, as well as the numbers of migrating and invading cells. A *p* < 0.05 was considered statistically significant. SPSS22.0 (IBM, Armonk, NY, USA) was used for statistical analysis and GraphPad software (GraphPad Software, La Jolla, CA, USA) was used to present the analyzed data.

**Table 1:** Correlation of high SPARC expression with clinical characteristics in 43 HCC.

**Groups**	**No**.	**SPARC**		**χ2**	***p*-value**
		**High expression**	**%**		
**Age**					
≥ 60 years	9	2	22.2%	0.067	0.795
<60 years	34	9	26.5%		
**Gender**					
Male	36	11	30.6%	2.874	0.090
Female	7	0	0%		
**GGT(IU/L)**					
≥ 30	30	9	30%	1.108	0.313
<30	13	2	15.4%		
**Child-Pugh class**					
A	26	9	34.6%	2.819	0.093
B	17	2	11.8%		
**NLR**					
≥ 3.7	13	3	23.1%	0.061	0.804
<3.7	30	8	26.7%		
**Tumor number**					
≥ 3	31	8	25.8%	0.003	0.957
<3	12	3	25.0%		
**Tumor diameter (cm)**					
≥ 5	12	4	33.3%	0.525	0.469
<5	31	7	22.6%		
**Lymph node metastasis**					
Positive	6	1	16.7%	0.291	0.590
Negative	37	10	27.0%		
**Portal vein tumor thrombosis**					
Positive	10	3	30.0%	0.134	0.715
Negative	33	8	24.2%		
**BCLC stage**					
A or B	29	8	27.6%	0.188	0.665
C	14	3	21.4%		
**TNM stage**					
II	26	7	26.9%	0.062	0.803
III or IV	17	4	23.5%		

*SPARC, Secreted protein acidic and rich in cysteine; HCC, hepatocellular carcinoma; GGT, γ-glutamyl transferase; NLR, Neutrophil-lymphocyte ratio; BCLC, Barcelona Clinic for Liver Cancer; TNM, Tumor, Node, Metastasis staging*.

## Results

### Detection of Expression of SPARC

**Figure 1:**
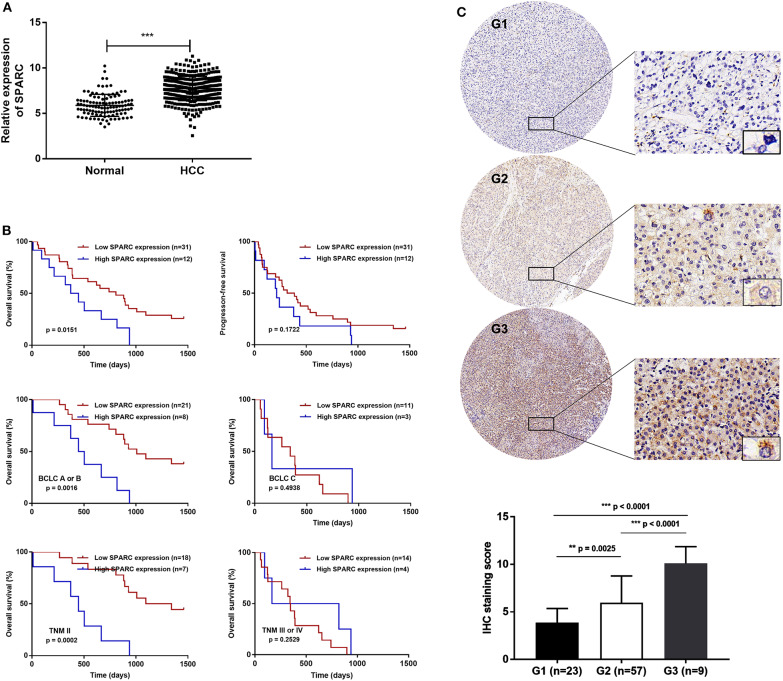
Expression of SPARC and its clinical significance in HCC patients. **(A)** Compared with healthy individuals, SPARC was highly expressed in the serum of patients with HCC in the TCGA dataset (*p* < 0.001). **(B)** There was an association between the serum expression of SPARC in patients with HCC receiving TACE and OS time. A cut-off value was implemented for dividing patients into a group with high expression (*n* = 12, serum SPARC ≥ 244 ng/mL) and a group with low expression (*n* = 31, serum SPARC <244 ng/mL). Kaplan-Meier analysis revealed that the OS of patients with high expression of SPARC might be poor (*p* = 0.0151). Both groups exhibited the same PFS (*p* = 0.1722). Regarding the cut-off value, as well as BCLC staging, we divided patients into the BCLC A/B low (*n* = 21) and high expression (*n* = 8) groups, and the BCLC C low (*n* = 11), and high expression (*n* = 3) groups, respectively. The OS of patients in both the BCLC A/B low and high expression groups with lower expression of SPARC appeared to be better (*p* = 0.0016). The cut-off value together with TNM grading were taken into consideration for dividing patients with TNM II high (*n* = 7) and low expression (*n* = 18) groups, and the TNM III/IV high (*n* = 4), and low expression (*n* = 14) groups, respectively. A higher serum level of expression of SPARC represented a shorter OS in the TNM II groups (*p* = 0.0002). **(C)** Cytoplasmic immunoreactivity (brown) and nuclear (blue) was observed. SPARC was significantly upregulated in higher grade human pathological HCC tissues. Immunohistochemical analysis (20 ×, and 400 × magnification) revealed an increased expression of SPARC in poorly- (G3, *p* < 0.0001; *n* = 9) compared with well-(G1, *p* < 0.0001; *n* = 23) and moderately- (G2, *p* = 0.0025; *n* = 57) differentiated HCC tissues. HCC, hepatocellular carcinoma; NOR, healthy individuals; TACE, transarterial chemoembolization; OS, overall survival; PFS, progression-free survival; BCLC, Barcelona Clinic Liver Cancer; TNM, Tumor Node Metastasis.

Bioinformatic analysis using TCGA dataset showed that the levels of SPARC in the plasma of patients with HCC were higher than that of healthy people (Figure 1A, *p* < 0.001). Table 1 illustrates the relation of the expression of SPARC to important clinical attributes of HCC. Based on these, the expression of SPARC was shown to not be related to any clinical parameters, such as age, liver function, tumor diameter, portal vein tumor thrombosis, BCLC stage, and TNM stage (Table 1).

### Survival Analysis

Evaluating the prognosis values exhibited by different variables assisted in finding factors that could predict the survival rate of patients with HCC undergoing TACE. Univariate analysis showed that expression of SPARC, neutrophil-lymphocyte ratio (NLR), tumor diameter, lymph node metastasis, portal vein tumor thrombosis, as well as BCLC and TNM staging exhibited a correlation with the OS of patients with HCC undergone TACE (*p* < 0.05, Table 2). Additionally, multivariate Cox regression analysis revealed the high expression of SPARC, portal vein tumor thrombosis (PVTT), and BCLC stage C as independent risk factors affecting prognosis (*p* < 0.05, Table 2).

**Table 2:** Univariate and multivariate analysis of prognosis factors for overall survival in HCC patients.

	**Univariate analysis**			**Multivariate analysis**		
	**HR**	***p*-value**	**95%CI**	**HR**	***p*-value**	**95%CI**
**SPARC expression**						
High vs.	2.194	0.036	1.051–4.582	2.331	0.033	1.072–5.071
Low						
**Age**						
≥ 60 years vs.	1.342	0.470	0.604–2.977			
<60 years						
**Gender**						
Male vs.	1.478	0.420	0.572–3.817			
Female						
**GGT(IU/L)**						
≥ 30 vs.	2.190	0.054	0.986–4.863			
<30						
**Child-Pugh class**						
A vs.	1.245	0.526	0.633–2.448			
B						
**NLR**						
≥ 3.7 vs.	2.132	0.035	1.056–4.303			
<3.7						
**Tumor number**						
≥ 3 vs.	1.503	0.281	0.717–3.152			
<3						
**Tumor diameter (cm)**						
≥ 5 vs.	2.764	0.007	1.326–5.760			
<5						
**Lymph node metastasis**						
Positive vs.	4.851	0.001	1.851–12.712			
Negative						
**Portal vein tumor thrombosis**						
Positive vs.	2.544	0.017	1.186–5.459	0.267	0.046	0.073–0.980
Negative						
**BCLC stage**						
A or B vs.	3.752	<0.001	1.811–7.773			
C				10.257	<0.001	3.043–34.579
**TNM stage**						
II or III vs.	3.723	<0.001	1.785–7.768			
IV						

*HR, hazard ration; CI, confidence interval*.

Taken into account the cut-off value, patients with HCC undergone TACE were divided into two groups, a group with high expression of SPARC, and a group with low expression of SPARC. Cut-off values at the 30th percentile were rounded as 244 ng/mL. The median survival period was 741 and 372 days in the group with low and high expression of SPARC, respectively. As revealed by the KM survival analysis, the 4-years OS of patients with a lower expression of SPARC was obviously higher (*p* = 0.0151, Figure 1B), whereas both groups had similar PFS at 4 years (*p* = 0.1722).

The expression of SPARC was also analyzed in relation to the BCLC and TNM staging of patients with HCC undergone TACE. Accordingly, the prognosis of patients with lower serum levels of SPARC was remarkably better at BCLC stage A or B and TNM stage II (*p* = 0.0016 and *p* = 0.0002). Regarding patients in BCLC stage C and TNM stage III or IV, the expression of SPARC did not show an obvious correlation to prognosis (*p* = 0.4938 and *p* = 0.2529).

To further explore the role played by SPARC in various grades of HCC pathological tissues, the protein levels of SPARC were evaluated in 89 collected HCC tissues. Immunohistochemical analysis revealed an increased expression of SPARC in poorly differentiated compared with well- and moderately-differentiated HCC tissues (*p* < 0.0001, *p* < 0.0001, and *p* = 0.0025, respectively; Figure 1C).

### Construction of SPARC-Downregulation and -Overexpression Models

**Figure 2:**
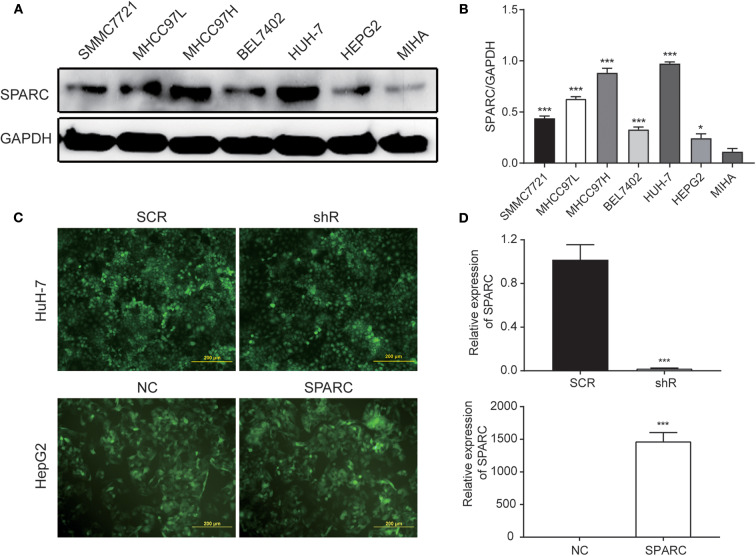
Cell infection. **(A,B)** Western blotting was performed to compare the expression of SPARC in HCC cell lines, namely SMMC7721, MHCC97L, MHCC97H, BEL7402, HUH-7, and HEPG2, as well as in the MIHA normal liver cell line. **(C,D)** Infection efficiency of lentivirus was expressed by green fluorescent. The efficiency of the 72 h infection with the shR and SPARC lentiviruses was found to be over 80%. Likewise, the efficiency of the knockdown and overexpression of SPARC following the 72 h infection with shR and SPARC lentiviruses were evaluated via RT-qPCR analysis, and found to be over 80%. *p < 0.05 and ***p < 0.001. SCR, scramble-sequence; NC, negative control; shR, SPARC-shRNA; SPARC, SPARC-infected.

We evaluated the expression of SPARC in six HCC cell lines, namely SMMC7721, MHCC97L, MHCC97H, BEL7402, HUH-7, and HEPG2, and in the MIHA normal liver cell line (Figures 2A,B). The SPARC-shRNA (shR) lentiviral construct was used to knockdown the expression of SPARC, whereas the SPARC construct was used to upregulate the expression of SPARC to form stable expression systems. For later related experiments, a SPARC down-regulation and a SPARC over-expression model were constructed using the HuH-7 and HepG2 cell lines, which have relatively high and low expression of SPARC, respectively (Figure 2C). The successful construction of these two models was confirmed by quantitative PCR analyses. Figure 2D present the significant downregulation and upregulation of the expression of SPARC in the shR and SPARC groups, respectively, relative to the SCR and NC groups.

### Knockdown of SPARC Suppressed the Progress of Tumor *in vivo*

**Figure 3:**
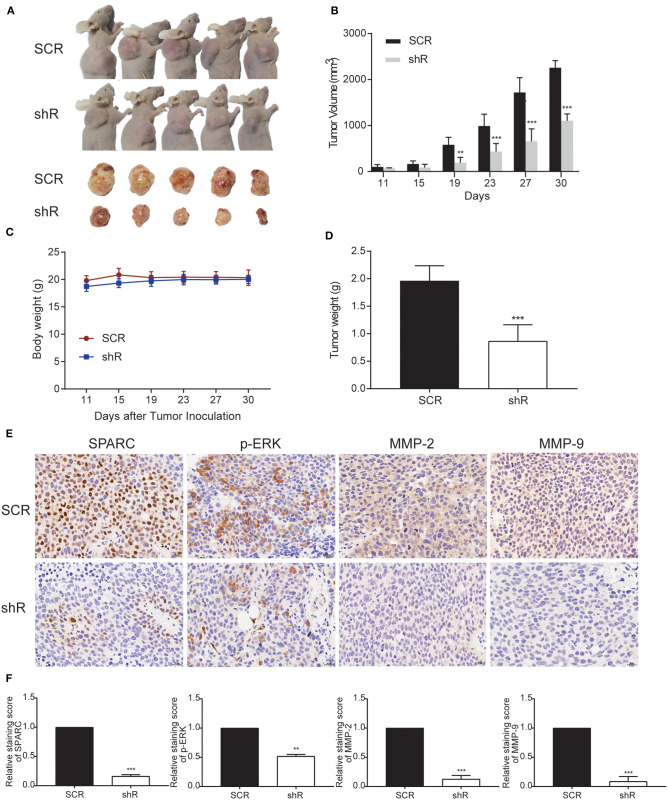
Downregulation of SPARC limits the growth of tumors *in vivo*. **(A)** BALB/C nude mice underwent a subcutaneous transplantation in their right upper sub-axillary with SCR or shR lentiviruses-infected HuH-7 cells (*n* = 5). The deficiency of SPARC suppressed the growth of tumors in nude mice 30 days after the transplantation. **(B)** The volume of tumors is expressed as the mean ± S.E.M. **(C)** Body weight. **(D)** Tumor weight. Immunohistochemistry (400 × magnification) analysis facilitated the detection of the expression levels of SPARC, p-ERK, MMP-2, and MMP-9 in tumor tissues **(E,F)**. **p < 0.01, and ***p < 0.001.

To further study the *in vivo* biological activity of SPARC, we developed a subcutaneous xenograft nude mice model. Collected HuH-7 cells (107 cells) infected with either shR or SCR plasmids were injected into the armpit of nude mice. Accordingly, as the expression of SPARC was downregulated in HuH-7 cells, the observed tumor size was remarkably smaller compared with the SCR group (Figures 3A,B). Data in Figure 3C showed that there was no significant difference in the body weight between the two groups. Knockdown of SPARC significantly decreased tumor weight in mice (Figure 3D). We performed immunohistochemistry (IHC) analysis to determine the protein levels of SPARC, p-ERK, MMP-2, and MMP-9 in the generated tumors. Accordingly, IHC analysis revealed much lower levels of SPARC protein in the shR relative to the SCR group. Likewise, IHC analysis demonstrated that tumors from the shR group presented much lower levels of p-ERK, MMP-2, and MMP-9 relative to the SCR group (Figures 3E,F).

### Downregulation of SPARC Weakened Malignant Behaviors of HCC Cells *in vitro*

**Figure 4:**
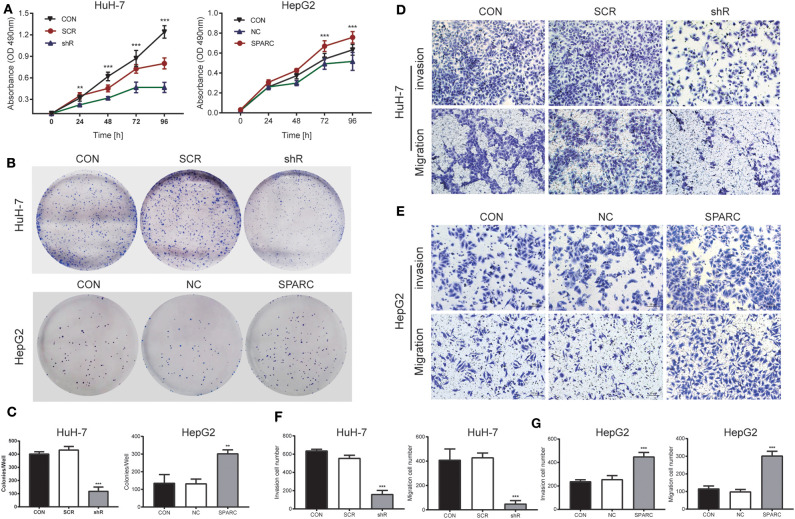
Knockdown of SPARC suppresses the proliferation, migration and invasion of HuH-7 cells, whereas upregulation of SPARC promotes those processes in HepG2 cells. **(A)** As shown by the MTT assay, 24 h of knockdown led to an obvious arrest of cells, whereas 72 h of upregulation greatly enhanced the proliferation of cells. **(B,C)** The plate colony formation assay facilitated the characterization of the ability of cells for colony formation. Assays on the migration and invasion of cells were conducted using shR or SCR-infected HuH-7 cells **(D,F)**, as well as SPARC or NC-infected HepG2 cells **(E,G)**. There was no significant difference of proliferation, migration and invasion between CON cells and SCR or NC cells. **p < 0.01, and ***p < 0.001. CON, untreated HuH-7 or HepG2 cell lines; SCR, scramble-sequence; NC, negative control; shR, SPARC-shRNA; SPARC, SPARC-infected.

As it was shown in the MTT assay, there was no significant difference of malignant behaviors between CON cells and SCR cells (*p* > 0.05). Downregulation of SPARC greatly inhibited the growth of cells after 24 h, relative to SCR cells (*p* < 0.01, Figure 4A). Similarly, the plate colony formation assay revealed that cells with lower levels of SPARC had smaller number and size of colonies relative to SCR-transfected cells (*p* < 0.001, Figures 4B,C). The migration and invasive ability of cells infected with SCR or shR for 24 h was analyzed and found to be remarkably limited owing to the downregulated expression of SPARC (*p* < 0.001 for both, Figures 4D,F).

To verify the results of our knockdown experiment, we designed a recovery experiment for detecting the biological functions possessed by SPARC in HepG2 cells with relatively low endogenous expression of the protein. Following 24 h of infection with SPARC, we observed an obvious increase in the proliferation of SPARC-infected cells after 72 h (*p* < 0.001, Figure 4A). Accordingly, cells with higher expression of SPARC exhibited a larger number and size of colonies relative to the NC-infected cells (*p* < 0.01, Figures 4B,C). Both the migration and invasion ability of cells infected with NC or SPARC for 24 h, SPARC group cells exhibited an obvious increase relative to NC group cells (*p* < 0.001 for both, Figures 4E,G). And there was no significant difference between CON group cells and NC group cells (*p* > 0.05).

### SPARC Facilitated Growth and Metastasis of HCC Cells via Activation of the ERK/MMPs Pathway

**Figure 5:**
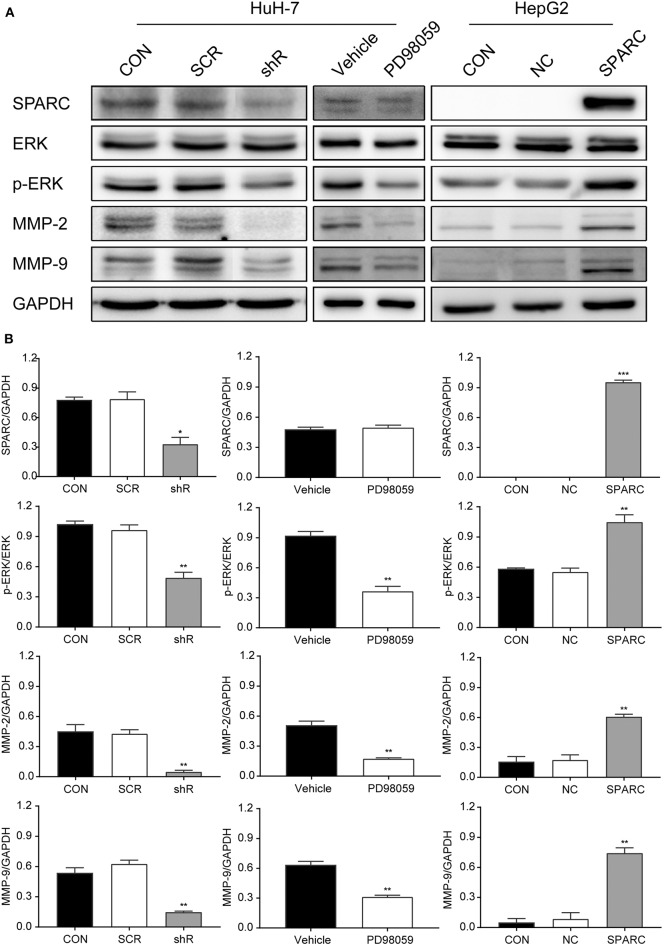
Knockdown of SPARC inhibits the ERK1/2-MMP2/9 pathway, suppressing the proliferation and metastasis of HCC cells. **(A)** Expression of proteins involved in the SPARC-ERK-MMPs pathway were assessed. **(B)** The gray value statistics of protein SPARC, p-ERK, MMP-2, and MMP-9. *p < 0.05, **p < 0.01, and ***p < 0.001. HCC, hepatocellular carcinoma.

Our study found that downregulation of SPARC for 72 h resulted in greatly inhibiting the ERK/MMPs pathway. The decrease in the expression of SPARC expression led to the downregulated expression of p-ERK1/2, MMP-2, and MMP-9 (Figure 5A). And the expression of SPARC, p-ERK1/2, MMP-2, and MMP-9 was not significantly different between CON and SCR (*p* > 0.05). To figure out the underlying mechanisms of this regulatory network, we aimed to evaluate the expression of the proteins involved in the downstream pathway by administering PD98059 (20 μM), an inhibitor of the p-ERK1/2 pathway, to HuH-7 cells. Following treatment of cells with PD98059 for 24 h, our results demonstrated a decrease in the expression of p-ERK1/2, MMP-2, and MMP-9 relative to those of the vehicle group (Figure 5A).

In contrast, the recovery experiment led to opposite results. Western blot analysis revealed that the levels of p-ERK1/2, MMP-2, and MMP-9 were remarkably increased after infection with SPARC for 72 h (Figure 5A). And the expression of SPARC, p-ERK1/2, MMP-2, and MMP-9 was not significantly different between CON and NC (*p* > 0.05). Analysis of the gray values of SPARC, ERK, p-ERK, MMP-2, as well as of MMP-9 contributed to the evaluation of the protein quantity, indicating an obvious change in the level of these proteins in the shR/PD98059/SPARC relative to the SCR/vehicle/NC group (Figure 5B).

## Discussion

The expert consensus statement of the 2010 International Hepato-Pancreato-Biliary Association defined TACE as a standard therapeutic approach for unresectable HCC, regardless of portal vein involvement (main portal vein excluded) (4). Despite delayed tumor progression and enhanced OS due to ischemic necrosis caused by arterial embolization, TACE could hardly achieve complete necrosis in the target lesion. Following TACE, incomplete embolization might result in intrahepatic or extracapsular tumor invasion. Therefore, it is of great significance to search for independent risk factors influencing the prognosis of patients with HCC before performing TACE.

As reported, SPARC was shown to promote the proliferation, and migration of tumor cells and correlate with the prognosis of HCC. Moreover, it was shown to help obtain stem cell phenotypes, as well as to facilitate the epithelial-mesenchymal transition of liver cancer cells, exhibiting a close association with the development of the tumor, its metastatic dissemination, and postoperative recurrence, as well as its resistance to radiotherapy or chemotherapy (13–15). However, it is necessary to further study the molecular mechanisms of the SPARC-related pathways in liver cancer.

Accordingly, this study aimed at confirming the value of SPARC for predicting prognosis in patients with HCC before TACE, as well as evaluating the mechanisms of action of SPARC during the development of HCC. The TCGA database revealed that SPARC exhibited an obviously higher expression in HCC relative to the healthy group. To further explore the potential value of SPARC in the preoperative prognosis of patients with HCC undergoing TACE, we quantified the expression of SPARC in the plasma of patients. Our study showed that increased expression of SPARC in the plasma of patients with HCC led to a shorter OS after TACE; however, high expression of SPARC did not remarkably affect PFS. Increased expression was also shown to cause shorter OS in BCLC stage A or B, and TNM grade II. As revealed by K-M analysis, the expression of SPARC in the plasma of patients with BCLC stage C, and TNM grade III, or IV was not related to OS. Hence, we suggested a negative correlation between the expression of SPARC before TACE and the prognosis of patients with HCC, especially those with BCLC stage A or B, and TNM stage II tumors. To clarify its mechanism, we further analyzed the relationship between the expression of SPARC and the grade of pathological tissues. The results indicated that the levels of expression of SPARC increased with the increasing grade of pathological tissues.

Furthermore, we investigated the preventive effect of SPARC in HCC, both *in vitro* and *in vivo*, using the HuH-7, and HepG2 human HCC cell lines, as well as HuH-7 xenografts in nude mice. Accordingly, when the expression of SPARC decreased, the viability, the colony formation ability and the migration and invasive ability of HuH-7 cells was greatly inhibited (Figure 4). Exogenous SPARC could intensify the proliferation of HCC cells with time. Our recovery experiments showed the ability of the overexpression of SPARC to remarkably promote the proliferation, migration, and invasion of HCC cells (Figure 4). These findings suggested that SPARC has a physiological role in promoting the growth of HCC cells and might be capable of facilitating the migration, as well as invasion of HCC cells. Likewise, in our *in vivo* model, the finding suggested that if the *in vivo* levels of SPARC are low, the expression of MMP-2/9 and p-ERK would be downregulated to suppress the formation and development of ectopic HCC cell tumor, thereby inhibiting the proliferation of the tumor (Figure 3). Combining all these findings, it could be suggested that SPARC might significantly affect the occurrence and development of HCC.

Tumor metastasis is one of the main factors limiting the efficacy of chemotherapeutic agents in patients with HCC. Intravasation and extravasation of HCC cells through basement membranes are essential steps in the metastatic cascade (16). The MMP proteins are known to act as an essential family of proteolytic enzymes participating in trophoblast invasion, and studies have shown that MMP-2 functions as an important enzyme in the degradation of type IV collagen during invasion (17, 18). As a member of the MMP family, MMP-9 has been reported to not only destroy type IV collagen, but also to lead to the degradation of extracellular matrix proteins, and to also help in the formation of distal metastases (19, 20). The ERK1/2 signaling pathway is known to essentially affect various cellular processes, such as migration, proliferation, and apoptosis (21). As reported, MMP-2 and MMP-9 can regulate the ERK signaling pathway, thereby promoting migration and invasion of cancer cells (22, 23). Noted, p-ERK was shown to be capable of affecting transcription factors, and regulating the transcription of many MMPs (24). This study showed that if phosphorylation of ERK1/2 was inhibited in HuH-7 and HepG2 cells, the activity of MMP-2 and MMP-9 would decrease, supporting the finding that p-ERK has the ability to regulate the expression of MMP-2/9.

As noted in all these processes, p-ERK and MMP-2/9 exhibited an obvious increase in their expression following knockdown of SPARC in HuH-7 cells, as well as in transplanted subcutaneous tumors. To further clarify the involvement of the proteins in this pathway, we evaluated their expression after treatment with a p-ERK inhibitor (PD98059, 20 μM). As revealed, the expression of p-ERK, and MMP-2/9 was significantly decreased after adding PD98059 to HuH-7 cells for 24 h. According to studies, increased expression of SPARC might lead to activation and subsequent phosphorylation of ERK1/2, with p-ERK regulating the transcription of MMP-2/9, thus promoting cell growth and proteolysis of extracellular matrix related to tumor invasion. In contrast, as shown in this study, knockdown of SPARC might result in suppressing the activation and phosphorylation of ERK1/2. Thereby, inactivation of ERK1/2 would downregulate MMP-2/9 at the surface of cell resulting in the inhibited development and invasion of tumor cells (Figure 5). Therefore, our findings suggested that SPARC could function in regulating the proliferation and metastasis of cells via the ERK/MMPs pathway and downregulation of SPARC could weaken the activity of both MMP-2 and MMP-9.

## Conclusions

To sum up, SPARC could regulate the ERK/MMPs pathway, thereby enhancing the proliferation and metastasis of tumor cells. Furthermore, SPARC could facilitate easier diagnosis of HCC, as well as identification of novel alternative targets specific to the management and treatment of HCC.

## Data Availability Statement

All datasets generated for this study are included in the article/supplementary material.

## Ethics Statement

The studies involving human participants were reviewed and approved by the ethics committee of Beijing Ditan Hospital. The patients/participants provided their written informed consent to participate in this study. The animal study was reviewed and approved by The Vital River Institutional Animal Care and Use Committee. Written informed consent was obtained from the owners for the participation of their animals in this study. Written informed consent was obtained from the individual(s) for the publication of any potentially identifiable images or data included in this article.

## Author Contributions

XianW and JL designed this study and supervised the entire process. LJ helped to collect tumor tissue from 89 patients. YaoL performed IHC and clinicopathologic data analyses, carried out the majority of *in vitro* experiments, and performed cell viability, and migration/invasion assay. YaoL wrote the manuscript. YF designed this study, helped to establish stable knockdown cells against SPARC and carried out the majority of *in vivo* experiments. XiaoW analyzed TCGA data and reviewed IHC slides. All authors were involved in critical review and discussion of this manuscript, read and approved the final manuscript.

## Conflict of Interest

The authors declare that the research was conducted in the absence of any commercial or financial relationships that could be construed as a potential conflict of interest.
